# The association between healthy beverage index and quality of life among overweight and obese women: a cross-sectional study

**DOI:** 10.1186/s12889-022-14501-1

**Published:** 2023-01-26

**Authors:** Niloufar Rasaei, Rasool Ghaffarian-Ensaf, Farideh Shiraseb, Melika Fallah, Fatemeh Gholami, Cain C. T. Clark, Khadijeh Mirzaei

**Affiliations:** 1 Department of Community Nutrition, School of Nutritional Sciences and Dietetics, Tehran University of Medical Sciences (TUMS), Tehran, Iran; 2 Department of Nutrition, Science and Research Branch, and Islamic Azad University, Tehran, Iran; 3 Centre for Intelligent Healthcare, Coventry University, Coventry, CV1 5FB UK

**Keywords:** Healthy beverage index, Quality of life, Diet, Overweight, Obesity

## Abstract

**Background:**

Although several studies have evaluated the association between patterns of beverage consumption with different components of quality of life separately, the findings are controversial. In addition, none have examined all components of quality of life together in relation to patterns of beverage consumption. Therefore, this study was conducted to identify the association between healthy beverage index (HBI) and quality of life among overweight and obese women.

**Methods:**

For this cross-sectional study, 210 obese and overweight women were recruited from health centers in Tehran, Iran. Using reliable and verified standard protocols, data on beverage intake, socio-demographic, physical activity, and anthropometric variables were assessed. Based on past studies, the predetermined HBI was estimated. Serum samples were used to determine biochemical characteristics, and quality of life was assessed using SF-36 questionnaires.

**Results:**

There was a significant association between total QoL score with T2 tertile of HBI in the adjusted model (β: 13.11, 95% CI: 1.52, 24.69, *p*-value = 0.027). General health had a significant negative association with T2 (β: -5.83; 95% CI: − 11.48, − 0.18; *p*-value = 0.043) and T3 (β: -6.20; 95% CI: − 12.37, − 0.03; *p*-value = 0.049). Women with greater adherence to the HBI had a higher physical functioning score, and there was a significant upward trend from the second to the third tertile (7.74 vs 0.62) (−trend = 0.036). There was a significant positive association between mental health with T3 of HBI (β: 4.26; 95% CI: 1.51, 5.98; *p*-value = 0.015) and a significant increasing trend was observed with increasing tertiles (P-trend = 0.045).

**Conclusion:**

In conclusion, there is a significant association between total QoL score, and its components, with HBI among overweight and obese women. However, additional well-designed studies are needed to confirm these findings.

## Introduction

Quality of life may be defined as an individual’s perception of his or her status in life, based on social and physical functioning, physical and emotional well-being, vitality, pain, and general health [1]. It seems that women, in comparison to men, tend to express lower quality of life [2]. In addition to this difference in gender, differences in quality of life score have been identified between obese and non-obese women [3]. Environmental factors, such as nutrition, can have major effects on quality of life [4], so different eating indices have been introduced to assess the quality of individual nutrition; for instance, to improve healthy beverage selection, the Healthy Beverage Index (HBI) was created [5]. Indeed, it has been shown that by adhering to the HBI, the risk of cardio metabolic disorders was lowered in US adults [5]. HBI can be used to detect the cumulative effects of multiple beverages instead of the small effect of a single beverage in relation to health outcomes [5]. It includes eight beverage categories, energy of total beverage and fluid consumption [5]. Consumption of beverages like milk, coffee, tea, and other unsweetened beverages can have different effects on general health [5]. Studies pertaining to different beverages and components of quality of life are scarce and conflicting. One study concluded that milk intake can affect weight status and body fat mass [6], while other has shown no association [7]. Fruit juice consumption may have a positive association with adiposity [8], while others failed to find a positive association between fruit juice and anthropometric indices [9, 10]. Water consumption may be an important strategy for prevention and treatment of obesity [11]; indeed, adequate fluid intake has been associated with better cognitive function [12] and blood glucose status [13]. Some studies expressed that sugar-sweetened beverage (SSB) consumption can increase the risk of blood pressure, overweight, diabetes, and cardiovascular diseases [14, 15], while one study showed that SSB is not associated to metabolic status [16]. Therefore, according to the equivocal nature of the literature and that no study has specifically assessed HBI in relation to quality of life in high-risk groups, such as overweight and obese women, we aimed to evaluate the association of the HBI with quality of life among obese and overweight Iranian women.

## Materials and methods

### Study population

This cross-sectional study was conducted using multi-stage simple random sampling and participants consisted of 210 women recruited from 20 Tehran Health Centers in 2018. Overall, 20 health centers were randomly selected from all health centers of the Tehran University of medical sciences. Sampling was such that, of people referred to Tehran health centers, if they met the inclusion criteria, they were randomly selected to enter the study. Adult women between the ages of 18 and 65 who had a body mass index (BMI) of 25 or above were eligible. Malignancies, cancer, liver disease, kidney disease, thyroid disease, cardiovascular disease, diabetes type I and II, menopause, pregnancy, lactation, smoking, any acute or chronic diseases, taking weight-loss supplements, going on a diet in the previous year, and taking drugs to lower blood pressure, glucose, and lipid levels in plasma were all exclusion criteria. Before the start of the trial, all participants signed a written informed consent form. The current study and informed consent were also accepted by the Tehran University of Medical Sciences (TUMS) local ethics committee, with the ethics number IR.TUMS.MEDICINE.REC. 1401.370. Based on the following formula, a sample size of 210 people was calculated as sufficient to evaluate the outcomes (both primary and secondary). Formula: *n* = (([Z_1-α_ + Z_1-β_) × √ 1-r ^2^]/r)^2^ + 2 which r = 0.25 [17], β =0.95, and α =0.05.

### Anthropometric measurements and body composition

We used a calibrated digital scale to measure each participant’s body weight, to the nearest 100 g, when they were not wearing shoes and wearing light clothing. While the participants were in a normal, standing position, we measured their height with a non-elastic tape with a precision of 0.5 cm. We divided the weight (in kilograms) by the square of the height to calculate the BMI (in kilograms per square meters). We utilized an elastic measuring tape with a precision of 0.5 cm to measure the waist circumference at the end of a natural exhale from the narrowest circumference of the waist. Using a strapless tape on the most noticeable, marked area, we measured the hip circumference. To decrease the measurement errors, all measurements were conducted by one person.

We used a bioelectrical impedance analyzer (BIA) (Inbody 770 Co., Seoul, Korea) to measure the body composition of all participants, following the manufacturer’s policy in terms of methodology, process, and precautions [18]. Inbody sensitivity and specificity values were 73 and 95.9%, respectively. The Inbody device calculates body fat percentage, fat mass, fat free mass, and predicted muscle mass on the basis of data obtained by Dual Energy X-ray Absorptiometry (DXA) using Bioelectrical Impedance Analysis (BIA). Participants were required to remove metal utensils, shoes, and extra clothes. It takes 15–20 seconds to measure weight, body mass index, and the body composition, including skeletal muscle mass, fat-free mass, fat mass, visceral fat, body fat percentage, and bone mineral content.

### Dietary assessment and HBI definition

We used a reliable and validated semi-quantitative standard food frequency questionnaire with 147 food items to assess all individuals’ regular dietary intake during the previous year [19].

The individuals were asked to report whether they consumed each food item on a daily, weekly, monthly, or yearly basis, based on the information provided in this questionnaire. The average size of each food item in the FFQ was explained to all participants during the face-to-face interview, and they were asked to rate how frequently they consumed each food item based on their standard unit on a daily, weekly, monthly, or yearly basis. We used the NUTRITIONIST 4 (Hearst Corporation, San Bruno, CA) food analyzer to convert the dietary intake data from the food frequency questionnaire into grams and milliliters and to evaluate the dietary intake data. The nutritionist 4 program was used to compute total energy, macronutrients, and micronutrients (Hearst Corporation, San Bruno, CA) [20].

Duffey and Davy [5] established a method to determine the HBI, where, similar to the Healthy Eating Index [21], the Healthy Beverage Index (HBI) can be used to assess the overall quality of beverage consumption and establish whether changes in consumption patterns are related to changes in health. According to the Beverage Guidance System, all beverages recorded as drank were divided into eight types; water, unsweetened coffee and tea, low-fat milk, diet drinks (including non-calorically sweetened coffee and tea and other artificially sweetened beverages), 100% fruit juice, alcohol (including beer, wine, and liquor), full-fat milk, and sugar-sweetened beverages (including fruit drinks, sweetened coffee and tea, and soda) were the eight categories of beverages consumed. The final HBI score ranges from 0 to 100, with a higher number indicating better beverage standard compliance and a healthy beverage consumption pattern [5]. The maximum final HBI score was 90 because diet drinks (with a score ranging from 0 to 5) and alcohol (with a score ranging from 0 to 5) were not consumed by our target group in this study. Liquids ingested as part of a meal (such as soup) were removed because the purpose of this study was to look into adherence to healthy beverage intake guidelines, rather than total fluid consumption. Our target audience did not eat these items, hence the maximum final HBI score was 90.

### Quality of life assessment

The SF-36 is a self-administered, short-form questionnaire that measures quality of life. It consists of 36 questions, 35 of which are compressed into eight multi-item scales, including: physical functioning (PF), role-physical (RP), bodily pain (BP), general health (GH), vitality (VT), social functioning (SF), role emotional (RE), and mental health (MH) [22].

(1) Physical Functioning (PF) is a 10-question scale that assesses a person’s ability to deal with daily physical demands such as personal hygiene, walking, and flexibility. (2) The Role-Physical (RP) scale is a four-item scale that assesses how much physical limitations hinder activity. (3) The Bodily Pain (BP) scale is a two-item measure that assesses the amount of discomfort felt in the previous four weeks and how much that pain interfered with routine work tasks. (4) The Overall Health (GH) scale is a five-item questionnaire that assesses personal perceptions of general health. (5) Vitality (VT) is a four-item scale that assesses a person’s sense of vigor, energy, and weariness. (6) Social Functioning (SF) is a two-item scale that assesses how much and how long physical health or emotional problems interfered with family, friends, and other social contacts in the last four weeks. (7) The Role-Emotional (RE) scale is a three-item questionnaire that assesses how much emotional problems interfere with work or other activities. (8) The Mental Health (MH) scale is a five-item questionnaire used to assess anxiety and depression symptoms [22, 23]. The SF-36 additionally includes a question about self-evaluating health changes over the previous year (reported health), which is not part of the eight categories, or the total SF-36 score. Each of these eight dimensions has a score ranging from 0 (worst health) to 100 (highest health).

### Biochemical assessment

Following an overnight fast, venous blood was drawn between 8:00 a.m. and 10:00 a.m. All of the samples were centrifuged, aliquoted, and stored at − 80 °C, and they were all evaluated using a single assay procedure. The enzymatic endpoint glycerol-3-phosphate oxidase–phenol 4-aminoantipyrine peroxidase (GPOPAP) was used to assess triglyceride (TG) and total cholesterol levels (TC). Low-density lipoprotein (LDL) and high-density lipoprotein (HDL) cholesterol were evaluated using a direct enzymatic clearance test. An immunoturbidimetric test using a Pars Azmoon kit was used to quantify serum hs-CRP levels (Pars Azmoon Inc. Tehran, Iran). The Nutrition and Biochemistry Laboratory of the TUMS School of Nutritional and Dietetics evaluated all samples using established methodologies.

### Assessment of IPAQ

Individuals’ physical activity was assessed using the short-term International Physical Activity Questionnaire (IPAQ) [24]. The physical activity of each participant over the previous seven days was calculated using this questionnaire. In 12 nations, the IPAQ questionnaires’ validity and reliability have been evaluated. The Spearman’s for this questionnaire’s criteria reliability was close to 0.8, whilst the median validity was reported to be around 0.30, which was consistent with findings from earlier validation studies. The IPAQ is a validated self-reported seven-item physical activity tool that records physical activity levels over the previous week (vigorous, moderate, walking, and inactive). The values are then multiplied by the corresponding metabolic equivalent (MET) quantities, and the resulting numbers are summed to determine the MET/min/week value.

### Statistical analysis

Statistical analyses were performed using SPSS software (version 26, SPSS Inc., Chicago, IL, USA) and *p* < 0.05 was set as statistically significant, while *P* = 0.05–0.07 was considered as marginally significant in the present study. The Kolmogorov-Smirnov test was used to determine the normality of data distribution; quantitative data were reported as means and standard deviation (SD), and categorical data were reported as numbers with percentages. According to the HBI, the participants were categorized to tertiles based on their score, to: tertile 1 (< 63), tertile 2 (63–67), and tertile 3 (> 67), respectively. The distribution of categorical factors (supplement use, educational status, job, income, and marriage) across Healthy Beverage Index groups was investigated using the Chi-square test. A comparison of the continuous variables including age, physical activity, anthropometric and body composition measurements, blood pressure, and biochemical variables, and dietary intake variables, including food groups, macronutrients and micronutrients across tertiles of the HBI was performed using analysis of variance (ANOVA), in addition to Bonferroni post hoc analysis where appropriate. Analysis of covariance (ANCOVA) was used for estimating energy-adjusted women’s dietary intakes across tertiles of a Healthy Beverage Index. Multivariate linear regression was performed for assessing the associations between Healthy Beverage Index and quality of life items in three different models: crude model; model 1, adjusted for energy intake, age and BMI; model 2, adjusted for model 1 plus education status.

## Results

### Study population

Overall, 210 participants were included in this study. Based on the HBI score, all participants were divided into tertiles such that the overall prevalence of them was 35.7% for tertile 1, 38.1% for tertile 2, and 26.2% for tertile 3, respectively. The mean (SD) age of participants was 36.09 (8.52) years, and the mean BMI of participants was 30.77 (4.22) kg/m^2^, whilst 40% of the participants used supplements. About 73% of the subjects were married, and 96.2% of them were employed. The economic status and education were such that 39% of the participants had a moderate economic status, and 48.1% of respondents were educated to bachelor level and higher. The mean (SD) of the total QoL score was 62.08 (29.05).

### General characteristics among tertiles of HBI

The general characteristics of study participants categorized based on the HBI tertiles are presented in Table 1. According to this table, *p*-values for all variables were reported in the crude and adjusted model (adjusting with age, BMI, energy intake, and physical activity). In the crude model, the mean difference of neck circumference (NC) (*P* = 0.022), total cholesterol (TC) (*P* = 0.009), high density lipoprotein cholesterol (HDL_C) (*P* = 0.012), and low-density lipoprotein cholesterol (LDL_C) (*P* = 0.001) were statistically significant. After controlling for confounding variables, the mean difference of physical activity (PA) (*P* = 0.045), height (*P* = 0.023), bone mineral content (BMC) (*P* = 0.009), skeletal muscle mass (SMM) (*P* = 0.020), waist to hip ratio (WHR) (*P* = 0.032), and triglyceride (TG) (*P* = 0.020) became significant. In terms of HDL_C and LDL_C, after controlling with potential cofounders, the mean difference remained significant. According to Bonferroni post-hoc testing, the significant mean difference in BMC was between T1 with T2 and T2 with T3 where this mean difference was higher in T1 compared to T2, and also the mean difference of the second tertile was lower than the third. In terms of the fat-free mass index (FFMI), HDL_C, and TG, the mean differences were higher in T1 compared to T2 except in HDL_C where the mean difference of the second tertile was higher. The mean differences of SMM and WHR were higher in the second tertile compared to the first one, and finally, the mean differences of IPAQ and LDL_C were between T2 with T3, where the mean difference of T3 was higher than T2 in IPAQ but in terms of LDL_C, T2 was higher than T3. In categorical variables, the education status was statistically significant after controlling for cofounders. There was no significant difference for other variables in Table 1 (Table 1).

**Table 1:** General characteristics among tertiles of HBI in obese and overweight women (*n* = 210)

Variables	Tertiles of HBI				
	T1 (< 63) ***N*** = 75	T2 (63–67) ***N*** = 80	T3 (> 67) ***N*** = 55	***p***-value	***p***-value^*^
**Demographic characteristic**					
Age (y)	35.72 (8.35)	36.73 (8.89)	36.01 (7.97)	0.749	0.658
IPAQ (MET min-week)	1012.44 (1936.43)	1122.00 (1198.99)	2029.30 (3693.09)^b^	0.051	**0.045**
**Anthropometric and body composition measurements**					
Weight (kg)	80.42 (11.16)	79.44 (13.61)	81.87 (10.60)	0.519	0.051
Height (cm)	161.90 (5.60)	160.69 (5.80)^c^	162.08 (6.09)	0.302	**0.023**
WC (cm)	93.60 (16.15)	94.39 (10.94)	99.16 (20.77)	0.161	0.101
NC (cm)	36.77 (2.16)	36.55 (2.08)	38.63 (3.20)	**0.022**	0.086
BMC (g)	2.73 (0.34)^a^	2.64 (0.37)^c^	2.69 (0.32)	0.244	**0.009**
SMM (%)	25.02 (3.32)	25.38 (3.67)^c^	25.84 (3.06)	0.493	**0.020**
SLM (%)	44.65 (5.26)	43.63 (5.85)	43.79 (5.20)	0.474	0.051
BMI (kg/m^2^)	30.66 (3.93)	30.82 (4.66)	30.94 (3.79)	0.932	0.740
WHR	0.92 (0.05)	0.93 (0.05)^c^	0.94 (0.04)	0.294	**0.032**
FFMI	18.04 (1.51)^a^	17.88 (1.62)	20.32 (17.73)	0.264	0.367
**Blood pressure**					
SBP (mmHg)	113.72 (11.47)	112.05 (14.14)	111.32 (14.24)	0.572	0.165
DBP (mmHg)	80.29 (8.44)	78.08 (11.60)	76.64 (9.30)	0.120	0.126
**Biochemical variables**					
TC (mg/dl)	178.51 (30.91)	183.55 (39.74)	198.56 (39.01)	**0.009**	0.118
TG (mg/dl)	132.34 (68.65)^a^	113.76 (54.40)	115.26 (58.77)	0.133	**0.020**
HDL_C (mg/dl)	44.05 (8.52)^a^	48.74 (11.28)	44.01 (13.05)	**0.012**	**0.005**
LDL_C (mg/dl)	98.81 (21.00)	97.26 (26.80)	82.05 (22.76)^b^	**0.001**	**0.008**
Hs-CRP (mg/l)	4.88 (5.04)	4.33 (4.67)	3.86 (4.05)	0.491	0.937
**Economic category**				0.156	0.199
Poor	28 (50.0)	16 (28.6)	12 (21.4)		
Moderate	28 (34.1)	36 (43.9)	18 (22.0)		
Good	17 (31.5)	20 (37.0)	17 (31.5)		
**Education category**				0.219	**0.001**
Illiterate	0 (0.0)	2 (66.7)	1 (33.3)		
Under diploma	5 (17.9)	15 (53.6)	8 (28.6)		
Diploma	27 (35.5)	30 (39.5)	19 (25.0)		
Bachelor and higher	42 (41.6)	32 (31.7)	27 (26.7)		
**Marital status**				0.765	0.582
Single	19 (34.5)	23 (41.8)	13 (23.6)		
Married	55 (35.9)	56 (36.6)	42 (27.5)		
**Supplement intake**				0.553	0.444
Yes	43 (51.2)	33 (39.3)	8 (9.5)		
No	27 (42.2)	30 (46.9)	7 (10.9)		
**Job category**				0.523	0.183
Employed	1 (50.0)	0 (0.0)	1 (50.0)		
Unemployed	74 (36.6)	76 (37.6)	52 (25.7)		

Values are represented as means (SD)

Categorical variables: N (%)

BMI considers as the collinear variable for anthropometrics and body composition variables

*BFM* Body Fat Mass, *BMC* Bone Mineral Content, *BMI* Body Mass Index, *DBP* Diastolic Blood Pressure, *FFMI* Fat-Free Mass Index, *HDL_C* High-Density Lipoprotein Cholesterol, *LDL_C* Low-Density Lipoprotein Cholesterol, *NC* Neck Circumference, *SBP* Systolic Blood Pressure, *SLM* Soft Lean Mass, *SMM* Skeletal Muscle Mass, *TC* Total Cholesterol, *TG* Triglyceride, *WC* Waist Circumference, *WHR* Waist to Hip Ratio, *hs CRP* High-Sensitivity C-Reactive Protein

ANCOVA (*p*-value*) was performed to adjust potential confounding factors (age, BMI, energy intake, physical activity)

*p*-values < 0.05 were considered as significant

^a^ significant difference was seen between T1 and T2

^b^ significant difference was seen between T1 and T3

^c^ significant difference was seen between T2 and T3

### Dietary intakes among tertiles of the HBI in obese and overweight women

Dietary intakes of all participants among tertiles of HBI are presented in Table 2. In the crude model, mean difference of linolenic acid (*P* = 0.001), vitamin E (*P* = 0.010), biotin (*P* = 0.036), whole grains (*P* = 0.001), fruits (*P* = 0.022), and red meat (*P* = 0.041) was statistically significant. After adjusting with energy intake, the mean difference of monounsaturated fatty acid (MUFA) (*P* = 0.019), carbohydrate (*P* = 0.017), Potassium (*P* = 0.016), vitamin B6 (*P* = 0.027), Pantothenic acid (*P* = 0.055), and vitamin C (*P* = 0.041) changed to significant. Linolenic acid, vitamin E, biotin, whole grains, and fruits remained significant after adjusting with cofounders. Other variables in Table 2 had no significant relationship (Table 2).

**Table 2:** Dietary intakes among tertiles of the HBI in obese and overweight women (*n* = 210)

Variables	Tertiles of HBI				
	T1 (< 63) ***N*** = 75	T2 (63–67) ***N*** = 80	T3 (> 67) ***N*** = 55	***p***-value	***p***-value^*^
	Mean (SD)				
**Dietary intakes**					
Energy (kcal/d)	2525.68 (670.80)	2678.56 (806.26)	2587.73 (814.74)	0.470	–
Protein (g/d)	86.08 (25.73)	92.96 (30.47)	88.07 (28.78)	0.320	0.684
Carbohydrate (g/d)	355.75 (116.08)	374.51 (126.96)	383.59 (129.04)	0.424	**0.017**
Total fat (g/d)	92.16 (31.28)	98.48 (33.73)	87.24 (32.16)	0.153	0.052
Cholesterol (mg/d)	238.35 (97.77)	270.12 (114.09)	260.90 (95.39)	0.162	0.387
SFA (mg/d)	26.65 (9.82)	30.29 (12.55)	26.09 (10.80)	0.059	0.063
MUFA (g/d)	31.83 (12.15)	31.71 (11.14)	28.17 (10.52)	0.147	**0.019**
PUFA (g/d)	20.50 (9.65)	20.17 (8.39)	17.84 (8.11)	0.210	0.095
Linolenic acid (g/d)	1.04 (0.59)	1.48 (0.67)	1.10 (0.52)	**0.001**	**0.001**
Linoleic acid (g/d)	18.05 (9.33)	17.04 (7.85)	15.24 (7.57)	0.181	0.069
EPA (mg/d)	0.03 (0.04)	0.03 (0.03)	0.03 (0.03)	0.720	0.596
DHA (mg/d)	0.12 (0.12)	0.10 (0.11)	0.11 (0.12)	0.719	0.585
TFA (mg/d)	0.0009 (0.002)	0.001 (0.002)	0.001 (0.004)	0.608	0.611
Sodium (mg/d)	4195.75 (1335.16)	4390.39 (1437.69)	3972.09 (1375.03)	0.249	0.194
Potassium (mg/d)	4110.79 (1566.16)	4412.91 (1472.64)	4719.56 (1665.52)	0.096	**0.016**
Vitamin A (RAE)	717.14 (349.91)	846.60 (425.03)	858.54 (507.79)	0.095	0.177
Β-carotene (mg/d)	4681.27 (2671.73)	5796.22 (3689.95)	5950.14 (4746.29)	0.089	0.160
Vitamin D (μg/d)	2.04 (1.62)	1.83 (1.34)	2.15 (1.63)	0.496	0.310
Vitamin E (mg/d)	19.35 (11.07)	15.20 (6.34)	15.54 (8.75)	**0.010**	**0.001**
Thiamin (μg/d)	2.04 (0.63)	2.08 (0.65)	2.03 (0.72)	0.882	0.406
Riboflavin (mg/d)	2.15 (0.87)	2.24 (0.73)	2.30 (0.95)	0.578	0.454
Niacin (mg/d)	24.01 (7.33)	26.18 (9.75)	25.39 (10.40)	0.343	0.723
Vitamin B6 (μg/d)	2.03 (0.64)	2.22 (0.75)	2.30 (0.76)	0.094	**0.027**
folate (μg/d)	588.44 (177.85)	609.71 (172.37)	621.76 (185.88)	0.559	0.293
Vitamin B12 (μg/d)	4.16 (1.91)	4.70 (2.45)	4.70 (3.40)	0.365	0.542
Biotin	36.85 (15.13)	38.08 (13.16)	44.82 (25.58)	**0.036**	**0.008**
Pantothenic acid	6.36 (2.18)	6.34 (1.89)	6.97 (3.67)	0.328	**0.055**
Vitamin K (μg/d)	185.35 (108.12)	243.77 (209.60)	258.04 (325.10)	0.125	0.178
Phosphorus (mg/d)	1609.31 (497.78)	1691.62 (534.58)	1638.43 (559.50)	0.630	0.989
Vitamin C μmol/L)	179.01 (159.25)	186.77 (101.70)	228.14 (127.78)	0.101	**0.041**
Calcium (mg/d)	1138.60 (416.04)	1197.82 (413.37)	1167.81 (424.00)	0.697	0.998
Iron (mg/d)	18.05 (5.90)	19.14 (5.93)	18.71 (6.88)	0.559	0.910
Magnesium (mg/d)	448.85 (145.78)	473.38 (147.65)	478.12 (164.87)	0.482	0.399
Zinc (mg/d)	12.80 (4.29)	13.40 (4.21)	13.09 (4.68)	0.701	0.948
Copper (mg/d)	1.89 (0.61)	2.03 (0.62)	2.12 (1.04)	0.217	0.082
Manganese (mg/d)	7.09 (2.43)	7.33 (2.79)	7.17 (3.73)	0.888	0.946
Selenium (mg/d)	121.04 (38.16)	120.16 (43.18)	116.07 (49.30)	0.802	0.216
Chromium (mg/d)	0.12 (0.08)	0.11 (0.09)	0.09 (0.09)	0.249	0.140
Total fiber (g/d)	42.75 (19.13)	45.83 (17.01)	44.65 (18.36)	0.531	0.767
Caffeine (mg/d)	140.73 (101.87)	174.46 (128.42)	176.28 (276.96)	0.396	0.458
**Food groups**					
Whole grains (g/d)	6.42 (7.77)	7.19 (11.07)	13.67 (15.01)	**0.001**	**0.001**
Fruits (g/d)	477.82 (372.87)	500.44 (300.18)	647.12 (401.28)	**0.022**	**0.006**
Vegetables (g/d)	418.82 (260.28)	461.96 (249.80)	507.20 (326.37)	0.205	0.250
Nuts (g/d)	13.02 (16.76)	18.08 (17.17)	15.91 (19.26)	0.215	0.461
Legumes (g/d)	53.80 (44.74)	55.47 (41.51)	54.89 (42.47)	0.972	0.697
Tea and coffee (ml/d)	681.17 (503.71)	855.23 (624.84)	903.13 (1399.35)	0.293	0.422
Refined grains (g/d)	441.31 (237.54)	409.66 (194.69)	425.89 (277.49)	0.712	0.219
Dairy (ml/d)	391.09 (244.07)	376.88 (212.09)	428.26 (324.62)	0.537	0.372
Eggs (g/d)	20.35 (12.89)	21.53 (13.26)	24.80 (14.96)	0.186	0.155
Fish and seafood (g/d)	12.78 (12.93)	11.30 (10.56)	13.15 (13.92)	0.657	0.435
White meat (g/d)	43.32 (34.69)	53.90 (53.55)	46.23 (33.31)	0.297	0.351
Red meat (g/d)	18.60 (14.69)	26.72 (22.71)	22.81 (20.43)	**0.041**	0.095

Values are represented as means (SD)

*DHA* Docosahexaenoic Acid, *EPA* Eicosapentaenoic Acid, *MUFA* Monounsaturated Fatty Acid, *PUFA,* Polyunsaturated Fatty Acid, *SFA* Saturated Fatty Acid, *TFA* Trans Fatty Acid

ANCOVA (*p* value*) was performed to adjusted potential confounding factors (energy intake)

*p*-values < 0.05 were considered as significant

### Total QOL score and its components among HBI tertiles

The association of total QoL score items among HBI tertiles were shown in Table 3**.** In the crude model, physical functioning (*P* = 0.002) and total QoL score (*P* = 0.031) were statistically significant. After adjusting for age, energy intake, and BMI in model 1, only physical functioning had a significant mean difference among the tertiles of HBI (*P* = 0.041). In model 2, after adjusting for age, energy intake, BMI, and education status, the mean difference of physical functioning (*P* = 0.036) remained statistically significant (Table 3).

**Table 3:** QoL items among HBI in obese and overweight women (*n* = 210)

Variables		Tertiles of HBI			
		T1 (< 63) ***N*** = 75	T2 (63–67) ***N*** = 80	T3 (> 67) ***N*** = 55	***p***-value
**General Health**	Crude	71.08 (16.12)	65.24 (18.40)	64.88 (17.71)	**0.073**
	Model 1	69.06 (2.26)	65.74 (2.10)	66.35 (2.55)	0.557
	Model 2	69.09 (2.27)	65.93 (2.13)	66.40 (2.57)	0.588
**Physical Functioning**	Crude	85.95 (14.59)	83.41 (18.40)	74.88 (19.40)	**0.002**
	Model 1	84.30 (2.19)	84.61 (2.04)	76.85 (2.48)	**0.041**
	Model 2	84.45 (2.19)	84.61 (2.06)	76.70 (2.48)	**0.036**
**Role Physical**	Crude	80.43 (39.49)	77.63 (41.94)	87.03 (33.90)	0.395
	Model 1	80.35 (5.21)	76.42 (4.85)	84.99 (5.90)	0.523
	Model 2	80.10 (5.23)	76.01 (4.90)	85.33 (5.92)	0.473
**Role Emotional**	Crude	78.26 (41.54)	68.14 (46.63)	66.66 (47.58)	0.278
	Model 1	80.12 (6.18)	65.49 (5.75)	67.41 (6.99)	0.213
	Model 2	80.07 (6.21)	64.98 (5.82)	67.40 (7.03)	0.202
**Social Functioning**	Crude	74.81 (20.12)	70.36 (28.48)	68.61 (22.18)	0.328
	Model 1	74.19 (3.00)	69.17 (2.79)	68.33 (3.40)	0.390
	Model 2	73.96 (2.99)	68.80 (2.80)	68.67 (3.39)	0.407
**Bodily Pain**	Crude	62.27 (20.42)	62.93 (22.11)	63.14 (22.30)	0.971
	Model 1	62.26 (2.91)	63.28 (2.71)	64.96 (3.29)	0.848
	Model 2	62.24 (2.92)	62.83 (2.73)	64.91 (3.30)	0.840
**Vitality**	Crude	71.07 (19.03)	69.94 (21.22)	68.00 (19.04)	0.695
	Model 1	71.23 (2.61)	70.46 (2.43)	68.84 (2.95)	0.848
	Model 2	70.85 (2.58)	70.09 (2.41)	69.19 (2.92)	0.925
**Mental Health**	Crude	77.10 (23.97)	73.40 (24.63)	68.91 (26.18)	0.195
	Model 1	75.39 (3.31)	73.75 (3.08)	70.83 (3.75)	0.695
	Model 2	75.17 (3.31)	73.33 (3.10)	71.10 (3.75)	0.753
**Health Transition Item**	Crude	42.02 (29.85)	45.33 (27.16)	46.29 (23.49)	0.647
	Model 1	39.44 (3.63)	47.35 (3.38)	49.57 (4.11)	0.172
	Model 2	39.43 (3.64)	46.92 (3.41)	49.56 (4.13)	0.178
**Total quality of Life**	Crude	58.28 (29.61)	72.01 (22.80)	60.53 (30.56)	**0.031**
	Model 1	57.49 (4.16)	70.48 (4.23)	61.69 (5.23)	0.090
	Model 2	57.44 (4.18)	70.55 (4.25)	61.68 (5.25)	**0.079**

Model 1: Adjusted for age, energy intake, BMI

Model 2: model further with education status

Data in crude model are presented as mean (SD)

Date in model 1 and model 2 are presented as mean (SE)

*QoL* Quality of Life

*p*-values < 0.05 were considered as significant

*P* value with unadjusted (crude)

### Association between HBI tertiles with total QoL score

Crude and adjusted β and 95% CI of total QoL score and its components across tertiles of HBI was shown in Table 4. In the crude model, there was a significant association between total QoL score with T2 tertile of HBI (β: 13.73, 95%CI: 3.25, 24.21, *p*-value = 0.010), in the model 2 adjustment (adjusted for age, energy intake, BMI, and education status), this significant association remained (β: 13.11, 95% CI: 1.52, 24.69, *p*-value = 0.027) and there was no significant trend based on tertiles in the crude model (P-trend = 0.463) or model 2 (P-trend = 0.429).

**Table 4:** Association between HBI tertiles with QoL and its components in obese and overweight women (*n* = 210)

Variables	Tertiles	B	SE	(95% CI)	***p***-value	P-trend
**General Health**						
Crude	T2	-5.83	2.88	(−11.48, −0.18)	**0.043**	**0.040**
	T3	-6.20	3.14	(−12.37, −0.03)	**0.049**	
Model 1	T2	−3.12	3.00	(−9.01, 2.77)	0.299	0.382
	T3	−2.97	3.57	(−9.98, 4.03)	0.406	
Model 2	T2	−2.97	3.01	(−8.89, 2.94)	0.324	0.386
	T3	−2.96	3.59	(−10.00, 4.06)	0.408	
**Physical Functioning**						
Crude	T2	−2.53	2.88	(−8.18, 3.11)	0.379	**0.001**
	T3	−11.07	3.15	(−17.25, −4.90)	**0.001**	
Model 1	T2	0.71	2.90	(−4.98, 6.40)	0.806	**0.042**
	T3	7.51	3.45	(1.28, 8.73)	**0.030**	
Model 2	T2	0.62	2.91	(−5.08, 6.32)	0.831	**0.036**
	T3	7.74	3.46	(0.53, 14.96)	**0.025**	
**Role Physical**						
Crude	T2	−2.80	6.44	(−15.43, 9.83)	0.664	0.392
	T3	6.60	7.04	(−0.001, 20.40)	0.089	
Model 1	T2	−3.55	6.89	(−17.07, 9.97)	0.607	0.636
	T3	4.42	8.20	(0.00, 20.50)	0.060	
Model 2	T2	−3.78	6.90	(−17.32, 9.76)	0.584	0.600
	T3	4.90	8.21	(0.00, 21.01)	0.060	
**Role Emotional**						
Crude	T2	−10.11	7.45	(−24.73, 4.50)	0.175	0.140
	T3	−11.59	8.15	(− 27.56, 4.38)	0.155	
Model 1	T2	−13.04	8.25	(−29.22, 3.12)	0.114	0.180
	T3	−12.52	9.81	(−31.76, 6.72)	0.202	
Model 2	T2	−13.42	8.28	(−29.65, 2.81)	0.105	0.186
	T3	−12.33	9.84	(−31.64, 6.96)	0.210	
**Social Functioning**						
Crude	T2	−4.45	3.98	(−12.27, 3.35)	0.264	0.145
	T3	−6.20	4.35	(−14.74, 2.33)	0.154	
Model 1	T2	−4.06	4.02	(−11.94, 3.81)	0.312	0.216
	T3	−5.80	4.78	(−15.18, 3.57)	0.225	
Model 2	T2	−4.27	4.00	(−12.12, 3.57)	0.285	0.249
	T3	− 5.32	4.76	(− 14.65, 4.01)	0.264	
**Bodily Pain**						
Crude	T2	0.66	3.56	(−6.31, 7.65)	0.851	0.817
	T3	0.87	3.89	(−6.75, 8.51)	0.822	
Model 1	T2	1.79	3.87	(−5.79, 9.37)	0.644	0.537
	T3	2.79	4.60	(−6.22, 11.82)	0.543	
Model 2	T2	1.42	3.87	(−6.15, 9.01)	0.712	0.537
	T3	2.83	4.60	(−6.18, 11.85)	0.538	
**Vitality**						
Crude	T2	1.12	3.28	(0.56, 7.31)	0.732	0.097
	T3	3.07	3.58	(1.10, 3.96)	**0.032**	
Model 1	T2	1.21	3.48	(−0.14, 6.60)	0.150	**0.037**
	T3	2.66	4.14	(0.78, 5.46)	0.021	
Model 2	T2	0.30	3.43	(−7.04, 6.43)	0.129	0.630
	T3	2.03	4.08	(0.05, 5.98)	**0.031**	
**Mental Health**						
Crude	T2	−3.70	4.09	(−11.73, 4.33)	0.367	**0.068**
	T3	8.18	4.47	(0.00, 9.59)	0.067	
Model 1	T2	−0.86	4.41	(−9.51, 7.77)	0.844	**0.041**
	T3	4.72	5.24	(0.009, 5.55)	0.068	
Model 2	T2	−1.09	4.39	(−9.71, 7.52)	0.803	**0.045**
	T3	4.26	5.22	(1.51, 5.98)	**0.015**	
**Health Transition Item**						
Crude	T2	3.30	4.50	(−5.52, 12.13)	0.463	0.371
	T3	4.26	4.90	(−5.35, 13.88)	0.085	
Model 1	T2	6.80	4.90	(−2.79, 16.41)	0.165	0.296
	T3	9.27	5.80	(2.10, 20.66)	**0.010**	
Model 2	T2	6.36	4.90	(−3.25, 15.98)	0.195	0.057
	T3	9.18	5.81	(2.20, 20.57)	**0.014**	
**Total QoL score**						
Crude	T2	13.73	5.34	(3.25, 24.21)	**0.010**	0.463
	T3	2.25	6.11	(−9.74, 14.24)	0.713	
Model 1	T2	12.99	5.90	(1.42, 24.56)	**0.028**	0.432
	T3	4.20	6.91	(−9.35, 17.76)	0.544	
Model 2	T2	13.11	5.90	(1.52, 24.69)	**0.027**	0.429
	T3	4.24	6.91	(−9.30, 17.80)	0.539	

Binary logistic regression was used

Tertile 1 consider as a reference group

Adjusted model 1: Adjusted for age, energy intake, BMI

Adjusted model 2: Adjusted for age, energy intake, BMI, education

*HBI* Healthy Beverage Index, *CI* Confidence Interval, *QoL* Quality of Life

*p*-values < 0.05 were considered as significant

P-trend < 0.05 were considered as significant

*P* value with unadjusted (crude)

In terms of general health in the crude model, there was a significant negative association in T2 (β: -5.83; 95% CI: − 11.48, − 0.18; *p*-value = 0.043) and T3 (β: -6.20; 95% CI: − 12.37, − 0.03; *p*-value = 0.049).

In the crude model, there was a significant negative association in term of the physical functioning component in T3 of HBI (β: -11.07; 95% CI: − 17.25, − 4.90; *p*-value = 0.001) and a significant decreasing trend was observed with increasing tertiles (P-trend = 0.001). In the model 1 adjustment (adjusted for age, energy intake, and BMI), we observed a significant positive association between physical functioning with T3 of HBI (β: 7.51; 95% CI: 1.28, 8.73; *p*-value = 0.030). A significant increasing trend with rising tertiles was observed (P-trend = 0.042). After controlling potential confounders in model 2, a significant positive association have shown between physical functioning with T3 of HBI (β: 7.74; 95% CI: 0.53, 14.96; *p*-value = 0.025), which means women with greater adherence of HBI had higher physical functioning score. There was a significant upward trend from the second to the third tertile (7.74 vs 0.62) (P-trend = 0.036).

There was a significant association between vitality and T3 of HBI in the crude model (β: 3.07; 95% CI: 1.10, 3.96; *p*-value = 0.032) and adjusted model 2 (β: 2.03; 95% CI: 0.05, 5.98; *p*-value = 0.031). In terms of health transition items, in model 2, we observed a significant relationship between this component and HBI tertiles (β: 9.18; 95% CI: 2.20, 20.57; *p*-value = 0.014). Finally, in model 2, we observed that there was a significant positive association between mental health with T3 of HBI (β: 4.26; 95% CI: 1.51, 5.98; *p*-value = 0.015) and a significant increasing trend was observed with increasing tertiles (P-trend = 0.045) (Table 4).

## Discussion

We sought to study the association between total QoL score and HBI among overweight and obese Iranian women. According to our findings, there was a significant association between total QoL score and its components with HBI among overweight and obese women. In this study, we combined all components of total QoL score, that, until now, had been investigated separately. Indeed, to our knowledge, this is the first study that investigate all aspects of total QoL score with HBI among overweight and obese women. Dietary intakes among tertiles of the HBI in obese and overweight women showed increasing trends for carbohydrate, MUFA, linolenic acid, potassium, vitamin E vitamin B6, biotin, pantothenic acid, vitamin C, whole grains, and fruits with increasing tertiles of HBI, after adjusting for energy. According to our results, QoL items among HBI in obese and overweight women showed that physical functioning, as a component of total QoL score, was associated with HBI. The association between HBI tertiles with QoL and its components in obese and overweight women indicated a significant increasing trend between physical functioning and mental health with increasing tertiles of HBI.

Dietary intakes among tertiles of the HBI in obese and overweight women showed increasing trends for carbohydrate, MUFA, linolenic acid, potassium, vitamin E vitamin B6, biotin, pantothenic acid, vitamin C, whole grains, and fruits across tertiles of HBI. These findings are concordant with a previous study which reported positive associations between higher HBI scores and more favorable health status because of the anti-inflammatory effect of HBI [5].

According to our results, physical functioning, as a component of total QoL score, was associated with HBI. In our analysis, after controlling for potential confounders, a significant increasing trend between physical functioning and mental health was observed with increasing tertiles of HBI. In terms of physical functioning, literature reports suggest that low HBI score is associated with poor physical functioning. Accordingly, consumption of less alcohol and sugar-sweetened beverages has been associated with better physical functioning [25]. Obesity is a factor that can affect physical functioning. Low HBI and consumption of beverages with high sugar content can contribute to obesity, and subsequently physical functioning [26]. Interestingly. A previous study on middle aged and older adults found that higher water intake can increase weight loss, and so can elicit better physical function [27]. Higher coffee consumption in older adults has been shown to be independently associated with better physical performance, which can be attributed to the caffeine, polyphenol, mineral, other bioactive ingredients of coffee [28].

Some studies investigated the association of mental health and health status with HBI. Indeed, in one study, it was shown that consumption of unhealthier beverages could cause poor mental health and health status in women. In this report, however, men’s mental health was less affected [29].

One of the principal mechanisms by which mental health affected by HBI is the higher glycemic load of beverages. Sugar can have an effect on oxidative stress and inflammatory processes which are linked to mental health [30, 31]. According to literature reports, consuming the beverages that help to have higher HBI score, like 100% fruit juices, is associated with lower prevalence of poor mental health [32]. In comparison to whole fat dairy consumption, low fat dairy products may exert more beneficial effects on social functioning, stress, and memory, and can contribute to better mental health. It has been posited that this association may be associated with higher saturated fatty acid intake, which can cause poorer mental health [33]. Based on the results of a prior study, consumption of coffee had no effect on QoL, but can affect mental health [34]. Other studies have shown that consuming high levels of coffee could positively impact on mental health [35, 36]. When caffeine blocks the adenosine receptors A1 and A2, adenosine will increase in the noradrenergic, cholinergic, dopaminergic, and serotoninergic systems. Following this, alertness, attention, wellness, and energy will increase [34]. Reports have shown that low HBI score is associated with low mood, where low HBI, by effecting neurotransmitters, could be associated with low mood [37–39]. Low consumption of water can have a negative effect on mood [40], indeed, when the consumption of water is low, the sympathetic nervous system function will decrease, which may confer a negative effect on mood by reducing the β-adrenergic receptors sensitivity [41].

We showed that vitality and health transition item had a significant association with HBI. A cross-sectional study showed moderate drinkers had a significantly higher vitality. In another study, it was demonstrated that moderated drinkers had better QoL score than abstainers [42–44]. A possible mechanism for the effect of alcohol on vitality is that the alcohol makes people more prone to exercise and then exhibit higher vitality [42]. However, we did not show any significant trend for vitality and health transition item based on HBI and our results were not consistent with these studies in this regard.

There are some limitations identified in this study that should be noted. First, this is a cross-sectional study, and thus, causality cannot be inferred, highlighting the need for prospective studies to confirm our findings. Second, this study was conducted only on women and therefore this sample do not represent the general population. Third, the sample size that we use to conduct this study was small, although following power analysis, our sample was shown to be sufficient. Fourth, we had time and budget constraints, impacting the length of study duration. Finally, because we used a self-report questionnaire for QoL, this study may be affected by information bias due to subjects misreporting, under reporting, or over reporting. Concomitant to the aforementioned limitations, it is important to mention the strengths of the present study. One the strengths is that we used an FFQ that validated in Iranian population. Moreover, this is the first study to have investigated the association of QoL and HBI among overweight and obese women. The results of our study can be generalized to all overweight and obese women in Tehran, which can be useful for public health. In this study we used and assessed all items of QoL, allowing insight into all facets of QoL. Finally, the present study was conducted in a developing country, where information about diet–disease associations is limited.

## Conclusion

Based on our findings, there may be a significant association between total QoL score and its components with HBI among overweight and obese women. These findings are important as they can be used to improve and inform approaches to increase total QoL score. Nevertheless, we recommend more studies should be conducted to reduce or account for the limitations of the present study.

## Data Availability

The data that support the findings of this study are available from correspond author but restrictions apply to the availability of these data, which were used under license for the current study, and so are not publicly available. Data are however available from the authors upon reasonable request and with permission of correspond author.
